# Methyl Jasmonate Orchestrates Multi-Pathway Antioxidant Defense to Enhance Salt Stress Tolerance in Walnut (*Juglans regia* L.)

**DOI:** 10.3390/antiox14080974

**Published:** 2025-08-08

**Authors:** Ruining Nie, Chengxu Wu, Xinying Ji, Ao Li, Xu Zheng, Jiajia Tang, Leyuan Sun, Yi Su, Junpei Zhang

**Affiliations:** 1 Research Institute of Forestry, Chinese Academy of Forestry, Beijing 100091, China; nieruining@163.com (R.N.); maoliwcx@163.com (C.W.); jixinying111@163.com (X.J.); a17637359516@163.com (A.L.); woshizhengxu2022@163.com (X.Z.); 19513324237@163.com (L.S.); suyii2002@126.com (Y.S.); 2 State Key Laboratory of Tree Genetics and Breeding, Beijing 100091, China; 3 Key Laboratory of Tree Breeding and Cultivation of the State Forestry and Grassland Administration, Beijing 100091, China; 4 Chongqing Academy of Forestry, Chongqing 400036, China; tangjiajia1989@126.com

**Keywords:** *Juglans regia* L., MeJA, NaCl, phenylalanine pathway, physiological indices, transcriptome

## Abstract

Walnut (*Juglans regia* L.), an ecologically and economically important species, requires the elucidation of its salt stress response mechanisms for improved salt tolerance breeding. This study elucidates the physiological and molecular mechanisms through which exogenous methyl jasmonate (MeJA) mitigates salt stress in walnut, providing novel strategies for salt-tolerant cultivar development. This integrated study combined physiological, biochemical, and multi-omics analyses to decipher how exogenous MeJA enhances ROS scavenging through the synergistic activation of phenylalanine (Phe), tryptophan (Trp), and α-linolenic acid pathways, establishing a multilevel antioxidant defense network. MeJA treatment effectively mitigated salt stress-induced oxidative damage, as demonstrated by a significant 16.83% reduction in malondialdehyde (MDA) content, concurrent 11.60%, 10.73% and 22.25% increases in superoxide dismutase (SOD), peroxidase (POD), and catalase (CAT) activities, respectively, the elevation of osmoregulatory soluble sugars (SS), and 1.2- to 2.0-fold upregulation of key antioxidant enzyme genes (SOD, POD, APX, GPX, DHAR) and elevated osmoregulatory substances (soluble sugars, SS). Improved photosynthetic parameters (*P*_n_, *G*_s_) and chlorophyll fluorescence efficiency (*F*_v_/*F*_m_) collectively indicated reduced oxidative stress (improved by 7.97–23.71%). Joint metabolomic-transcriptomic analyses revealed MeJA-enhanced ROS scavenging via the coordinated regulation of Phe, Trp, and α-linolenic acid pathways. In summary, MeJA significantly enhanced reactive oxygen species (ROS) scavenging efficiency and comprehensive antioxidant capacity in walnut seedlings through the synergistic regulation of key metabolic pathways, effectively mitigating salt stress. These findings establish a crucial mechanistic foundation for understanding plant salt stress responses and advance the utilization of MeJA-mediated strategies for the genetic improvement of salinity tolerance in walnut. Future research should prioritize optimizing MeJA application protocols and functionally validating key regulatory genes for breeding applications.

## 1. Introduction

Soil salinization represents a critical challenge to global agricultural sustainability, with salinized soils exceeding 954 million hectares worldwide and expanding annually by 1–1.5 million hectares [1]. Under salt stress, plants endure three primary stresses—ionic toxicity, osmotic imbalance, and oxidative damage—where ROS burst-induced oxidative damage constitutes the core mechanism of cellular dysfunction. High salinity reduces soil water potential, impedes water uptake, and disrupts photosystem II electron transport, reducing photosynthetic efficiency while promoting ROS accumulation. These reactive species oxidize membrane lipids, proteins, and nucleic acids, inducing membrane lipid peroxidation (e.g., malondialdehyde (MDA) accumulation) that culminates in cell death [2,3]. Thus, elucidating plant ROS scavenging and antioxidant defense networks is critical for advancing salt tolerance.

Methyl jasmonate (MeJA), a key plant stress signaling molecule, has emerged as a research focus in salt tolerance studies due to its efficacy in regulating antioxidant defense. Studies demonstrate that MeJA modulates salt stress through dual mechanisms: direct enhancement of enzymatic antioxidant systems and coordinated regulation of non-enzymatic antioxidant networks. Exogenous MeJA significantly increases superoxide dismutase (SOD), peroxidase (POD), and catalase (CAT) activities, effectively scavenging ROS under salt stress. For instance, in *Nitraria tangutorum* Bobrov, MeJA enhanced antioxidant enzyme activities and reduced MDA content through SOD and POD gene upregulation [4]. Peng et al. [5] observed that soybean (*Glycine max* (L.) Merr.) flavonoids function as potent non-enzymatic antioxidants that directly quench ROS, whereas lignin forms physical barriers limiting ROS transport diffusion via enhanced cell-wall mechanical strength [6]. Furthermore, MeJA stimulates melatonin synthesis through the tryptophan pathway, which directly scavenges ROS while activating SOD/POD activity [7]. Additionally, MeJA-activated jasmonic acid-isoleucine (JA-Ile) from the α-linolenic acid pathway interacts with abscisic acid (ABA) and auxin (IAA) signaling to inhibit lipoxygenase (LOX)-mediated lipid peroxidation, thereby reducing ROS production at its source [8].

Walnut (*Juglans regia* L.), as a globally significant woody oilseed species cultivated across Mediterranean Europe, western Asia, and North America—regions increasingly affected by soil salinization—enhancing walnut salt tolerance has urgent international agricultural relevance. Salt-induced ROS accumulation compromises photosynthetic function, inhibits root development, and constrains cultivation [9]. Salt-induced oxidative damage in walnut manifests at ≥50 mM NaCl, characterized by ROS accumulation and photosynthetic decline [2]. Consequently, we employed 50 mM NaCl as the standardized stress level—a concentration widely adopted in plants [10] that reliably induces ionic/oxidative cascades while permitting MeJA efficacy assessment. The antioxidative function of externally applied methyl jasmonate has been well characterized in annual crops such as tomato (*Solanum lycopersicum* L.) and pomaceous species including apple (*Malus domestica* Borkh.) [11,12], but its function in walnut—particularly the mechanism coordinating key metabolic pathways to regulate ROS homeostasis—remains uncharacterized. Moreover, elucidating the synergistic actions of these pathways in ROS scavenging, ROS generation suppression, and membrane protection remains challenging [13]. The primary objective of this study was to elucidate the mechanism through which exogenous methyl jasmonate (MeJA) enhances salt tolerance in walnut seedlings. We employed integrated physiological, biochemical, transcriptomic, and metabolomic analyses to decipher MeJA-mediated synergistic regulation of key metabolic pathways (e.g., antioxidant biosynthesis, osmotic adjustment, hormone signaling), establish a multi-level antioxidant network responsible for improved ROS scavenging capacity under salt stress, provide a mechanistic framework for applying MeJA to enhance salt resilience in perennial woody plants, and propose an application framework for leveraging MeJA to improve salt resilience in economically vital woody plants across saline-vulnerable regions worldwide.

## 2. Materials and Methods

### 2.1. Plant Materials and Treatments

The experiment was performed in a controlled greenhouse at the Chinese Academy of Forestry Sciences (Beijing, 40°00′10″ N, 116°14′38″ E; altitude: 61 m). The plant material used was *Juglans regia* L. ‘Wen185’, a locally dominant cultivar provided by The Germplasm Resource Bank of the Jiamu Experimental Station, Xinjiang Academy of Forestry. Seeds were germinated in a substrate mixture of peat moss, perlite, and vermiculite (3:1:1, *v*/*v*/*v*), with standard fertilization and irrigation during seedling growth. Seedlings of uniform size and vigor were selected and transplanted into 20 L hydroponic containers (40 × 30 × 23 cm), with four seedlings per container. Continuous aeration was maintained using air pumps to ensure optimal plant growth.

A pre-cultivation gradient acclimation protocol was applied. Seedlings were irrigated with Hoagland nutrient solution at 1/8, 1/4, and 1/2 concentrations for 5 days each. Based on preliminary trials, NaCl and MeJA treatments were set at 50 mM and 50 μM, respectively. MeJA was dissolved in ethanol and co-applied with NaCl in the nutrient solution. CK groups received equivalent volumes of distilled water. The experimental design comprised eight treatment groups (Table 1), with 32 plants per group. Leaf and root tissues were sampled at 24 h and 7 days post-treatment initiation for physiological and histological analyses.

### 2.2. Measurement of Morphological Observation and Stomatal Indexes

Six uniformly sized seedlings were randomly selected from each treatment group. The seedlings were deactivated by heating at 105 °C for 20 min, followed by drying at 80 °C until constant mass was achieved to measure aboveground and root biomass.

Leaf samples (approximately 1 cm^2^) were collected from the 1st–2nd pair of functional leaves on the 2nd–3rd compound leaves below the shoot apex. The samples were sectioned into 0.5–1 mm^2^ fragments and fixed in 2.5–4% glutaraldehyde solution under vacuum infiltration until tissue sinking, followed by storage at 4 °C. Dehydration was performed using a Leica EM CPD300 automatic critical point dryer. Sputter coating with gold-palladium was conducted using a Leica EM ACE600 coater (Leica Microsystems, Wetzlar, Germany). Samples were imaged at ×400 and ×1800 magnifications using a Hitachi Regulus 8230 cold field-emission scanning electron microscope (Hitachi, Tokyo, Japan). Stomatal dimensions (length, SL, and width, SW; both in μm) were quantified from epidermal imprints at 1800× magnification using ImageJ 2.0 software (NIH, Bethesda, USA), and the SW/SL ratio was calculated.

### 2.3. Determination of Photosynthetic Parameters

Photosynthetic pigment content was measured according to the method described by Zhu et al. [14]. Photosynthetic parameters, including net photosynthetic rate (*P*_n_), stomatal conductance (*G*_s_), transpiration rate (*T*_r_), and intercellular CO_2_ concentration (*C*_i_), were determined using an LI-6400XT portable photosynthesis system (LI-COR Biosciences, Nebraska, USA). Measurements were conducted between 09:00 and 11:00 under clear-sky conditions, targeting the 1st–2nd pair of functional leaves on the upper-middle compound leaves. The following settings were applied: CO_2_ concentration = 400 μmol mol^−1^, photosynthetic photon flux density = 1000 μmol m^−2^ s^−1^, standard leaf chamber (2 × 3 cm), open gas exchange mode, and flow rate = 400 μmol s^−1^.

Chlorophyll fluorescence was measured with a Yaxin-1162 handheld fluorometer (Yaxinliyi, Beijing, China). Prior to measurements, leaves were dark-adapted for 30 min using leaf clips to determine the maximum quantum yield of PSII (*F*_v_/*F*_m_).

### 2.4. Determination of Oxidative Stress Biomarkers

Malondialdehyde (MDA) content was quantified using the thiobarbituric acid (TBA) method [15]. Soluble sugar (SS) levels were measured via the anthrone colorimetric assay [16]. An amount of 0.1 g of the plant sample was weighed, ground, and mixed with 5 mL of 50 mM phosphate buffer (pH 7.8). The mixture was then centrifuged for 10 min at 10,000 rpm and 4 °C, and the supernatant was taken as the enzyme extraction solution. The supernatant was collected and stored at 4 °C for the determination of SOD, POD, and APX activities [17,18]. Endogenous phytohormones, including indole-3-acetic acid (IAA), abscisic acid (ABA), salicylic acid (SA), and jasmonic acid (JA), were quantified using enzyme-linked immunosorbent assay (ELISA) kits (Jiangsu Jingmei Biotechnology, Jiangsu, China). All assays were performed according to manufacturer protocols with absorbance measured at 450 nm. Tissue extracts were diluted 1:5 (*v*/*v*) in provided buffer prior to analysis. Specific catalog numbers: IAA (JM-01121P1), ABA (JM-01051P1), SA (JM-09813P1), JA (JM-01103P1).

### 2.5. RNA Isolation and Transcriptomics Analysis

Total RNA was isolated from walnut leaves using an MJZol Total RNA Extraction Kit (Shanghai Majorbio Bio-Pharm Technology Co., Ltd., Shanghai, China) following manufacturer protocols, with integrity assessed via an Agilent 5300 Bioanalyzer and concentration quantified by NanoDrop ND-2000. High-integrity RNA samples (OD_260/280_ = 1.8–2.2; OD_260/230_ ≥ 2.0; RIN > 6.5; 28S:18S ≥ 1.0) with minimum yields of 1 μg (or >10 ng for low-input protocols) were used for library preparation. Libraries were sequenced on NovaSeq X Plus (Illumina, PE150, NovaSeq Reagent Kits) or DNBSEQ-T7 (MGI, PE150, DNBSEQ-T7RS v3.0 Kit). Gene expression was quantified as FPKM, with DEGs identified by DESeq2 (|log2FC| ≥ 1, FDR < 0.05) or DEGseq (|log2FC| ≥ 1, FDR < 0.001). Functional annotation included gene ontology (GO) enrichment via Goatools and KEGG pathway analysis using a hypergeometric test (implemented in SciPy v1.10.0). Transcriptomic data were analyzed using the Majorbio Cloud Platform (www.majorbio.com or cloud.majorbio.com), “URL (accessed on 25 May 2025)”.

### 2.6. Metabolome Analysis

Frozen samples (100 mg dry weight) were pulverized using a cryogenic homogenizer (Wonbio-96c, operated at −10 °C, 50 Hz for 6 min), followed by ultrasonic-assisted extraction (5 °C, 40 kHz, 30 min). After equilibration (−20 °C, 30 min) and centrifugation (13,000× *g*, 4 °C, 15 min), the supernatant was analyzed via UHPLC-Q Exactive MS (Thermo Scientific, Massachusetts, USA) equipped with an ACQUITY UPLC BEH C18 column (100 × 2.1 mm, 1.7 μm; column temperature: 40 °C). Chromatographic separation was achieved using Mobile phase A: 0.1% (*v*/*v*) formic acid in ultrapure water and Mobile phase B: 0.1% (*v*/*v*) formic acid in HPLC-grade acetonitrile (flow rate: 0.3 mL/min; gradient elution). MS detection used dual-polarity modes (spray voltage: 3.5 kV; capillary: 320 °C). Quality control employed pooled QC samples. Raw data processing (Progenesis QI v3.0) included noise removal and internal standard filtration, with normalized data annotated using the Majorbio Cloud Platform (www.majorbio.com or cloud.majorbio.com), “URL (accessed on 20 June 2025)”.

### 2.7. Real-Time Quantitative PCR Assay

Total RNA was extracted from 100 mg frozen leaf tissue using the Polysaccharide Polyphenol Total RNA Extraction Kit (Tiangen Biotech, Beijing, China). cDNA synthesis and RT-qPCR were performed with the RR037Q Reverse Transcription Kit (TaKaRa Bio, Shiga, Japan) and TB Green Premix Ex Taq II (TaKaRa Bio, Shiga, Japan), respectively, using a LightCycler 480 instrument (Roche, Basel, Switzerland). Gene expression was quantified via the 2^−ΔΔCt^ method using *JrGAPDH* as the housekeeping gene. Primers were designed using NCBI Primer-BLAST, and their sequences are listed in Table S1.

### 2.8. Statistical Analysis

Triplicate biological replicates are expressed as mean ± SD. Treatment effects were evaluated using one-way analysis of variance (ANOVA) in SPSS 25.0, followed by Duncan’s multiple range test for post hoc comparisons (α = 0.05). Visualization was conducted in Origin 2021.

## 3. Results

### 3.1. Concentration Screening of MeJA

Seedlings were treated with different concentrations of MeJA under 50 mM NaCl stress, with 50 μM being the most effective. The results showed that 50 μM MeJA had the most significant alleviating effect on NaCl stress. The growth of seedlings treated with 50 μM MeJA was significantly improved compared to NaCl treatment, and the chlorophyll levels, as measured by SPAD value and Tchl, increased by 15.75% and 6.34%, respectively. Meanwhile, the MDA content in leaves was reduced by 17.70%, while SS content increased by 3.49% (Table S2). Therefore, 50 μM MeJA was selected for the experiment.

### 3.2. Effects of Exogenous MeJA on the Biomass and LRWC Under Salt Stress

**Figure 1:**
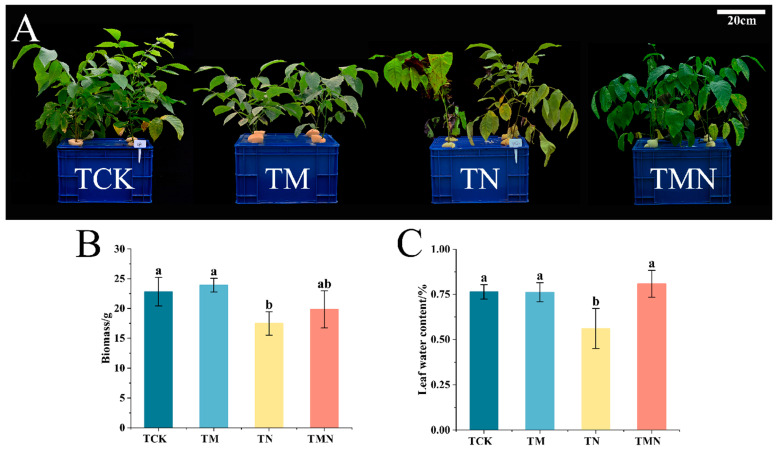
Effects of exogenous MeJA on walnut seedlings under salt stress. (**A**) Phenotype. (**B**) Biomass. (**C**) Leaf relative water content (LRWC). Data: means ±SE (*n* = 3). Lowercase letters indicate significant differences (*p* < 0.05, LSD test). TCK: Hoagland’s solution; TM: CK + 50 μM MeJA; TN: CK + 50 mM NaCl; TMN: CK +50 μM MeJA + 50 mM NaCl.

To investigate the mitigating effect of MeJA on walnut salt stress, the growth index of walnut seedlings was measured after 7 days of treatment. The addition of MeJA alleviated leaf yellowing and wilting compared to TN treatment (Figure 1A). The biomass of the TMN treatment was significantly higher than the TN treatment, showing an increase of 134.17% (Figure 1B). Additionally, the relative water content of leaves in the TMN treatment increased by 44.56% compared to the TN treatment (Figure 1C).

### 3.3. Effects of Exogenous MeJA on the Stomatal Characteristics, F_v_/F_m_, Photosynthetical Pigments and System Under Salt Stress

**Figure 2:**
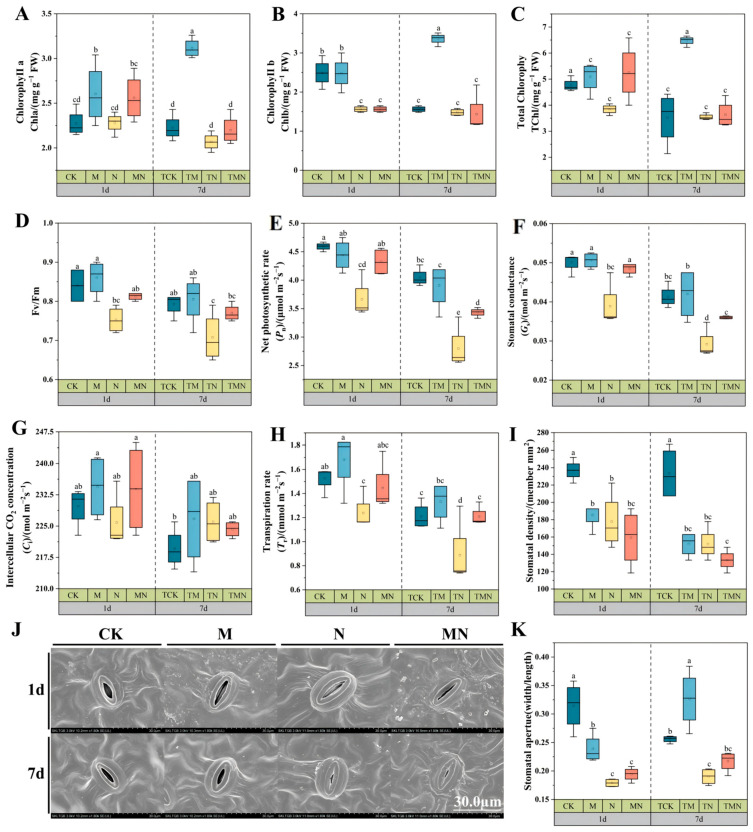
Exogenous MeJA enhances photosynthetic function in salt-stressed walnut seedlings. (**A**) Chlorophyll a content; (**B**) Chlorophyll b content; (**C**) Total chlorophyll content; (**D**) *F*_v_/*F*_m_; (**E**) Net photosynthetic rate; (**F**) Stomatal conductance; (**G**) Transpiration rate; (**H**) Intercellular CO_2_ concentration; (**I**) Stomatal density; (**J**) Stomatal morphology (1800×); (**K**) Stomatal width/length. Data: mean ± SE (*n* = 3). Lowercase letters indicate significant differences (*p* < 0.05, LSD) per time point. CK: Hoagland’s solution; M: CK + 50 μM MeJA; N: CK + 50 mM NaCl; MN: CK +50 μM MeJA + 50 mM NaCl.

Salt stress significantly inhibited the synthesis of photosynthetic pigments in walnut leaves. Compared with CK, the contents of Chla, Chlb, and TChl decreased by 0.7%, 59.61%, and 24.00%, respectively, 1 day after N treatment. After 7 days, Chla and Chlb in TN decreased by 7.61% and 14.86% versus TCK, while TChl change was insignificant (Figure 2A–C). Exogenous MeJA alleviated this inhibition: At 1 day: MN increased Chla, Chlb, and TChl by 12.28%, 72.43%, and 36.63% versus N. At 7 days: TMN increased Chla and TChl by 6.28% and 2.33% versus TN, while Chlb improvement was insignificant. M/TM treatments consistently showed the highest pigment contents (Figure 2A–C).

Salt stress severely impaired photosynthesis: At 1 day: N reduced *Pn*, *Gs*, and *Tr* by 25.36%, 29.13%, and 23.38% versus CK; *Ci* was unchanged. At 7 days: TN further reduced *Pn*, *Gs*, and *Tr* by 44.52%, 41.37%, and 36% versus TCK, while *Ci* increased by 2.93% (Figure 2E–H). MeJA significantly improved performance: At 1 day: MN increased *Pn*, *Gs*, and *Tr* by 18.05%, 23.71%, and 16.77% versus N. At 7 days: TMN increased these values by 22.73%, 24.13%, and 76.39% versus TN. *Ci* showed minimal differences across treatments (Figure 2E–H). Salt stress reduced photochemical efficiency: N decreased *F*_v_/*F*_m_ by 11.62% (1 day) and 12.85% (7 days) versus CK/TCK. MeJA effectively mitigated this: MN increased *F*_v_/*F*_m_ by 7.97% versus N at 1 day. TMN increased *F*_v_/*F*_m_ by 10.00% versus TN at 7 days (Figure 2D).

### 3.4. Effects of Exogenous MeJA on the Antioxidant Enzymes Activity, MDA, Soluble Sugar, and Endogenous Hormones Under Salt Stress

**Figure 3:**
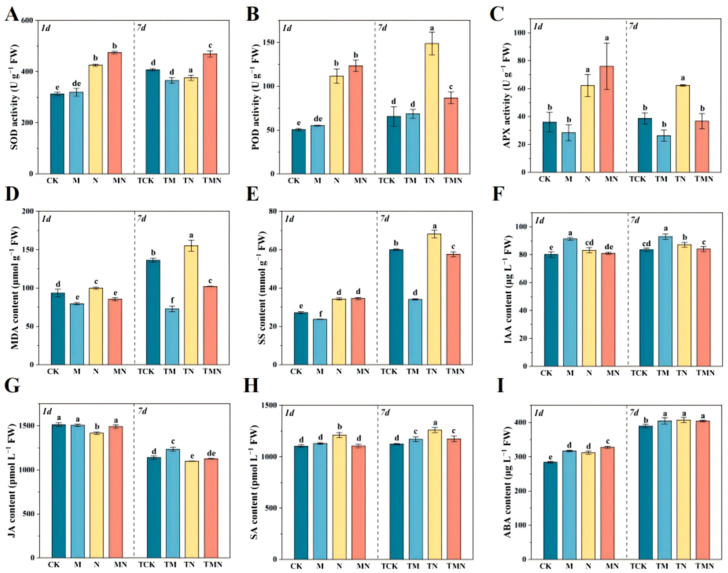
Exogenous MeJA modulates antioxidant and hormonal responses in salt-stressed walnut seedlings. (**A**) Superoxide dismutase (SOD) activities; (**B**) Peroxidase (POD) activities; (**C**) Ascorbate peroxidase (APX) activities; (**D**) Malondialdehyde (MDA) contents; (**E**) Soluble sugar (SS) contents; (**F**) Indole-3-acetic acid (IAA) contents; (**G**) Jasmonic acid (JA) contents; (**H**) Salicylic acid (SA) contents; (**I**) Abscisic acid (ABA) contents; Data: mean ± SE (*n* = 3). Lowercase letters indicate significant differences (*p* < 0.05, LSD) per time point. CK: Hoagland’s solution; M: CK + 50 μM MeJA; N: CK + 50 mM NaCl; MN: CK +50 μM MeJA + 50 mM NaCl.

The role of MeJA in mitigating salt stress was investigated through changes in antioxidant enzyme activities, soluble sugars, and MDA content. At 1 day, MeJA significantly enhanced antioxidant enzyme activities: MN increased SOD, POD, and APX by 11.60%, 10.73%, and 22.25%, respectively, versus N (Figure 3A–C). By 7 days, TMN maintained elevated SOD activity (24.90% higher than TN), while POD and APX decreased by 70.77% and 69.09% versus TN. Notably, although salt stress transiently increased MDA at 1 day, MN reduced it by 16.83% versus N, with no significant change in soluble sugar (SS). At 7 days, TMN further decreased MDA and SS by 52.2% and 18.24% relative to TN (Figure 3D,E).

The role of MeJA in hormone regulation was analyzed through IAA, JA, SA, and ABA dynamics. Under N stress at both time points, IAA showed an increasing trend but decreased insignificantly in MN; the highest IAA occurred in M (91.31 μg/L at 1 day) and TM (92.94 μg/L at 7 days) (Figure 3F). JA decreased under N/TN stress but increased insignificantly in MN (1489.25 pmol/L) and TMN (1126.66 pmol/L) (Figure 3G). For SA, N/TN elevated content, but MN and TMN caused significant reductions to 1105.59 pmol/L and 1173.15 pmol/L, respectively; M/TM showed no change versus CK/TCK (Figure 3H). ABA surged significantly in N (311.88 μg/L) at 1 day and further increased in MN (327.57 μg/L), while at 7 days, TM, TN, and TMN showed no significant differences (Figure 3I).

### 3.5. Differential Expression, GO and KEGG Pathway Annotation and Enrichment Analysis

**Figure 4:**
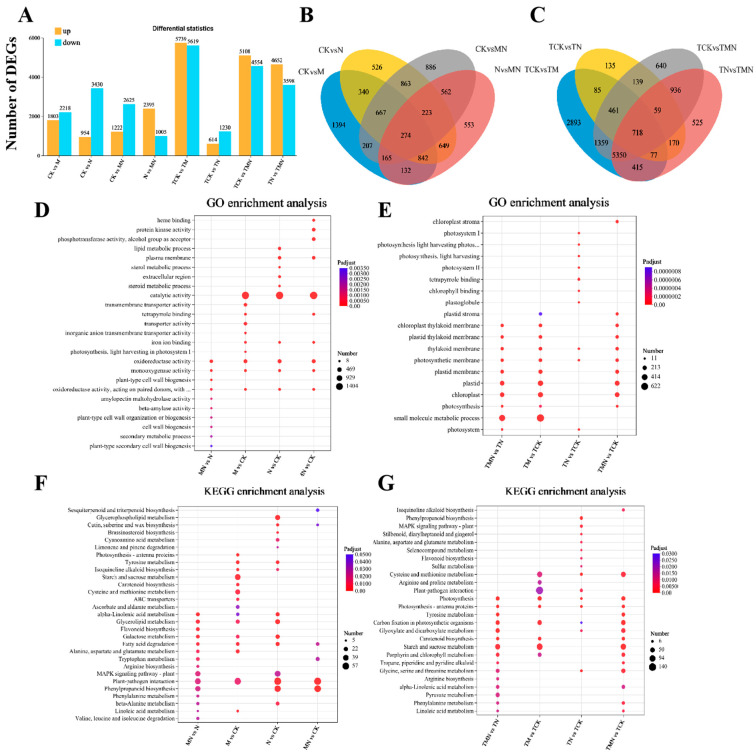
Multi-group comparative transcriptomics reveal MeJA-mediated salt stress responses. (**A**) DEG counts (up/down-regulated). (**B**,**C**) Venn diagrams of DEG overlap. (**D**,**E**) Top 10 enriched GO terms. (**F**,**G**) Top 10 enriched KEGG pathway. Comparison groups: 1d: MN vs. N, CK vs. M, CK vs. N, CK vs. MN; 7d: TMN vs. TN, TCK vs. TM, TCK vs. TN, TCK vs. TMN. Treatments: CK/TCK: Control; M/TM: 50 μM MeJA; N/TN: 50 mM NaCl; MN/TMN: NaCl + MeJA.

To examine the transcript levels of MeJA-treated walnut and its relationship with salt stress tolerance, we performed transcriptome sequencing on 24 samples. A total of 164.97 Gb of clean data was obtained, with each sample yielding more than 6.01 Gb. The average Q20, Q30, and GC content were more than 99.11%, 97.03%, and 44.88%, respectively, indicating high sequencing quality and reliable results. Based on the sequencing of each library, DEGs were identified for each of the two treatment comparisons. A total of 15,780 DEGs were identified from the analysis. Figure 4A shows the differences in DEGs between the eight treatments. We detected 4021, 4387, 3847, and 3400 DEGs associated with the four walnut leaf libraries of CK vs. M, CK vs. N, CK vs. MN, and N vs. MN (1d), respectively. Of these, 1803, 954, 1222, and 2395 were up-regulated DEGs. A total of 274 genes were differentially expressed across these four treatments. Similarly, 11,358, 1877, 9662, and 8250 associated DEGs were detected in the four libraries of TCK vs. TM, TCK vs. TN, TCK vs. TMN, and TN vs. TMN (7d), with 5739, 614, 5108, and 4652 up-regulated DEGs, respectively. A total of 718 genes were differentially expressed in these four treatments. These genes were associated with the response to exogenous MeJA, the response to NaCl, and the alleviation of NaCl stress by MeJA. The genes related to the alleviation of NaCl stress by MeJA were considered important candidates for further studies.

To elucidate the molecular mechanisms of MeJA-induced salt stress tolerance, gene ontology (GO) and Kyoto Encyclopedia of Genes and Genomes (KEGG) enrichment analyses were performed on the top 10 significantly enriched terms (Figure 4C–F). The results showed that in the GO enrichment analysis, oxidoreductase activity and monooxygenase activity were commonly enriched in the four treatment groups at 1 day. In addition, secondary metabolic processes, plant secondary cell wall biogenesis, and other terms were enriched in N vs. MN, while chloroplasts, cysts, and photosynthetic membranes were common enrichment terms among the three groups at 7 days: TN vs. TMN, TCK vs. TMN, and TMN vs. TCK (Figure 4C).

In the KEGG enrichment analysis, tryptophan metabolism, fatty acid degradation, and phenylpropane biosynthesis were commonly enriched pathways in N vs. MN and CK vs. MN at 1 day. Additionally, MAPK signaling pathway-plant, phenylalanine metabolism, linoleic acid metabolism, and degradation of valine, leucine, and isoleucine were enriched in N vs. MN. At 7 days, starch and sucrose metabolism, phenylalanine metabolism, α-linolenic acid metabolism, linoleic acid metabolism, photosystems, and carbon fixation by photosynthetic organisms were common pathways in TN vs. TMN and TCK vs. TMN (Figure 4E,F).

### 3.6. DEGs Involved in Key Metabolic Pathways

**Figure 5:**
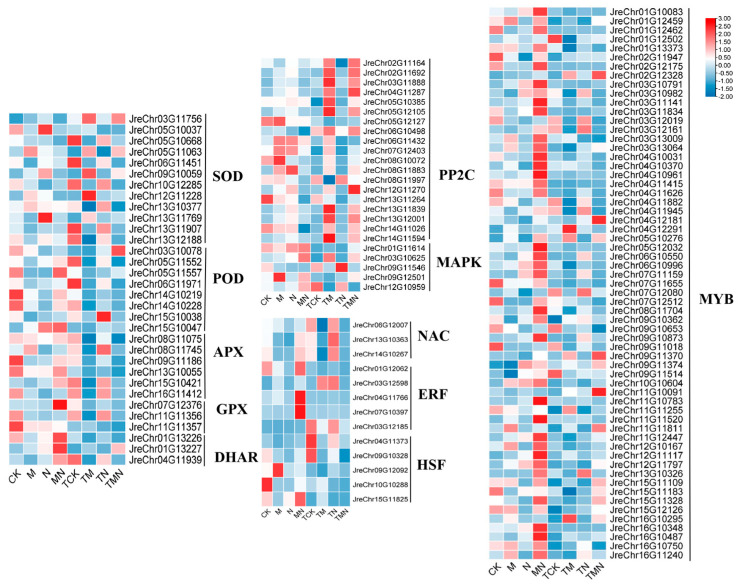
MeJA coordinates antioxidant genes and stress signaling transcriptomes to enhance salt tolerance in walnut.

Integrating physiological indicators with transcriptome data, we identified differentially expressed genes (DEGs) regulated by exogenous MeJA. Given the centrality of the antioxidant system in mitigating ROS accumulation, MeJA-induced DEGs were predominantly enriched in antioxidant pathways. Compared to NaCl treatment alone, MeJA supplementation downregulated SOD-related genes but significantly upregulated POD, APX, GPX, and DHAR expression. Concurrently, the MAPK signaling pathway—critical for abiotic stress perception and metabolic reprogramming—exhibited pronounced differential expression: MAPK cascade genes were upregulated, whereas PP2C genes (negative regulators) were downregulated. Furthermore, transcription factors (TFs) contributed substantially to salt stress responses, with exogenous MeJA activating key TF families (*ERF*, *HSF*, *MYB*) under NaCl stress (Figure 5).

### 3.7. Metabolomics Analysis

**Figure 6:**
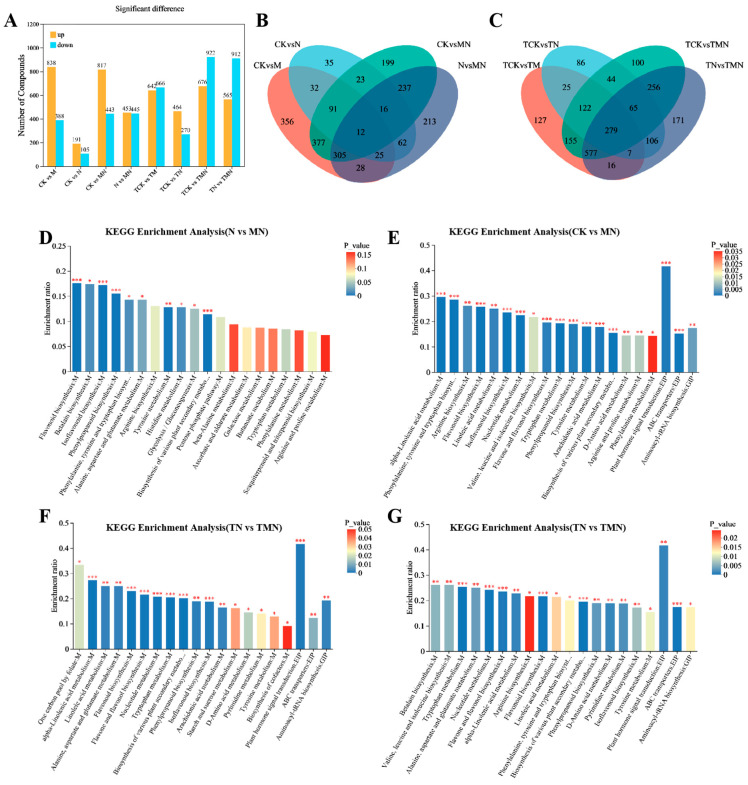
Metabolic reprogramming induced by MeJA under salt stress revealed by KEGG enrichment. (**A**) DAM counts (up/down-regulated). (**B**,**C**) Venn diagrams of DAM overlap. (**D**–**G**) Top 20 enriched KEGG pathways. Comparison groups: 1d: MN vs. N, CK vs. M, CK vs. N, CK vs. MN; 7d: TMN vs. TN, TCK vs. TM, TCK vs. TN, TCK vs. TMN. Treatments: CK/TCK: Control; M/TM: 50 μM MeJA; N/TN: 50 mM NaCl; MN/TMN: NaCl + MeJA. Significance levels *, ** and *** represent *p* < 0.05, *p* < 0.01 and *p* < 0.001.

To investigate the protective mechanisms of MeJA against NaCl stress in walnut, we performed a large-scale metabolomic profiling using ultra-high-performance liquid chromatography-tandem mass spectrometry (UHPLC-MS/MS). Applying stringent criteria (|log_2_FC| ≥ 1, adjusted *p* < 0.05, VIP > 1), 2666 significantly altered metabolites were classified as differentially accumulated metabolites (DAMs). To decipher metabolic responses to salt stress under MeJA, DAMs were analyzed across control groups (Figure 6A). We detected 1226, 493, 1260, and 896 relevant DAMs in the four walnut leaf libraries of CK vs. M, CK vs. N, CK vs. MN, and N vs. MN (1d), respectively. Of these, 838, 191, 817, and 453 were up-regulated DAMs, resulting in a total of 12 DAMs differentially expressed across the four treatments. At 7 days post-treatment, 1308, 734, 1598, and 1477 associated DAMs were detected in the four libraries TCK vs. TM, TCK vs. TN, TCK vs. TMN, and TN vs. TMN, respectively. Among these, 642, 464, 676, and 565 were up-regulated DAMs, resulting in a total of 279 DAMs differentially expressed across the four treatments. At 1 day post-treatment, the CK vs. M, CK vs. N, CK vs. MN, and N vs. MN groups exhibited 1226 (838 upregulated), 493 (191), 1260 (817), and 896 (453) DAMs, respectively, with 12 shared DAMs. At 7 days post-treatment, the TCK vs. TM, TCK vs. TN, TCK vs. TMN, and TN vs. TMN groups showed 1308 (642), 734 (464), 1598 (676), and 1477 (565) DAMs, respectively, with 279 shared DAMs.

Analysis of the top 20 KEGG-enriched pathways across four comparative groups (CK vs. MN, N vs. MN, TCK vs. TMN, TN vs. TMN) revealed significant enrichment in key metabolic pathways. These included phytohormone signaling, flavonoid–isoflavonoid metabolism, phenylalanine metabolism, tryptophan metabolism, linoleic acid metabolism, and α-linolenic acid metabolism. Notably, the phytohormone signaling pathway exhibited the highest enrichment levels in the CK vs. MN, TCK vs. TMN, and TN vs. TMN groups. This underscores its central role in MeJA-mediated stress responses (Figure 6).

### 3.8. Combined Analysis of Transcriptome and Metabolome

Integrated transcriptomic and metabolomic analyses were employed to elucidate the mechanisms underlying MeJA-mediated salt stress alleviation. KEGG co-enrichment analysis of transcriptome and metabolome data across four treatment groups (CK vs. MN, N vs. MN, TCK vs. TMN, TN vs. TMN) identified key regulatory pathways. Phytohormone signaling was significantly enriched in both transcriptomic and metabolomic profiles of the CK vs. MN and TCK vs. TMN groups (*p* < 0.05). Tryptophan and phenylalanine metabolism pathways were co-enriched in the CK vs. MN and N vs. MN comparisons, while α-linolenic acid metabolism was co-enriched in CK vs. MN and TN vs. TMN groups (Figure S1).

### 3.9. Effect of Exogenous MeJA on Phenylalanine Metabolism

Phenylalanine, an aromatic amino acid, serves as a critical precursor for plant secondary metabolite biosynthesis. Through the phenylpropanoid pathway, it generates diverse secondary metabolites, including lignans, flavonoids, and coumarins, which play essential roles in abiotic stress tolerance. Under salt stress (N), exogenous MeJA application markedly upregulated the phenylpropanoid pathway.

**Figure 7:**
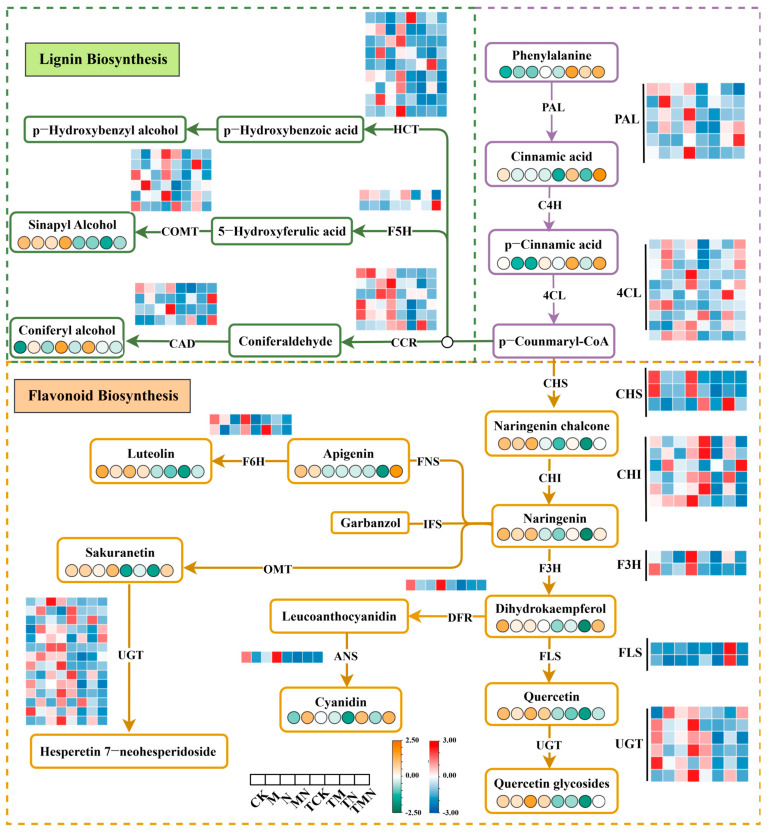
Integrative omics reveals MeJA-mediated synergistic regulation of phenylalanine metabolism under salt stress.

Compared to the N treatment, the MN group exhibited significant upregulation of core phenylalanine metabolism genes (PAL, C4H, 4CL), flavonoid biosynthesis enzymes (CHI, F3H, UGT), and lignin synthesis regulators (HCT, COMT, CCR). Concurrently, phenylalanine-derived core metabolites (phenylalanine, cinnamic acid, coumaric acid) and phenylpropanoid-derived hormones were significantly elevated by 7 days post-treatment. Notably, flavonoid precursors (chalcone, naringenin, quercetin) and the lignin metabolite erucitol showed even greater accumulation at 1 day post-treatment (Figure 7).

### 3.10. Effect of Exogenous MeJA on Tryptophan Metabolism

Tryptophan, an essential aromatic amino acid in plants, serves dual roles as both a protein building block and a precursor for phytohormones such as indole-3-acetic acid (IAA). Tryptophan metabolism plays a crucial role in regulating plant growth and stress adaptation. Under NaCl stress, exogenous MeJA application dynamically modulated tryptophan metabolic pathways.

**Figure 8:**
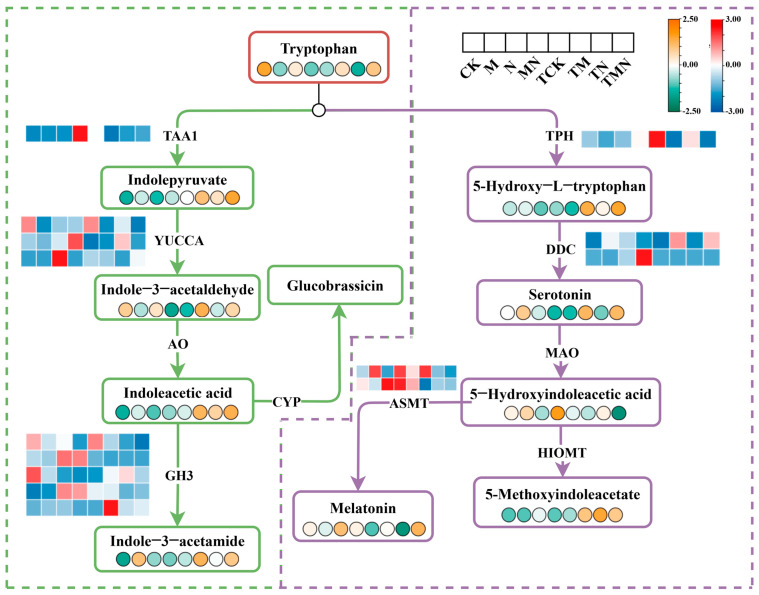
Integrative omics reveals MeJA-enhanced tryptophan metabolism reprogramming under salt stress.

Compared to the N group, MN treatment significantly upregulated key tryptophan metabolism enzymes (TAA1, ASMT). Metabolite profiling revealed time-dependent shifts. IAA synthesis intermediates (tryptophan, indole-3-acetaldehyde, IAA) decreased at 1 day post-treatment but rebounded by 7 days post-treatment. Serotonin precursors (5-hydroxytryptophan) and melatonin surged exclusively at 7 days post-treatment (Figure 8).

### 3.11. Effect of Exogenous MeJA on α-Linolenic Acid Metabolism

**Figure 9:**
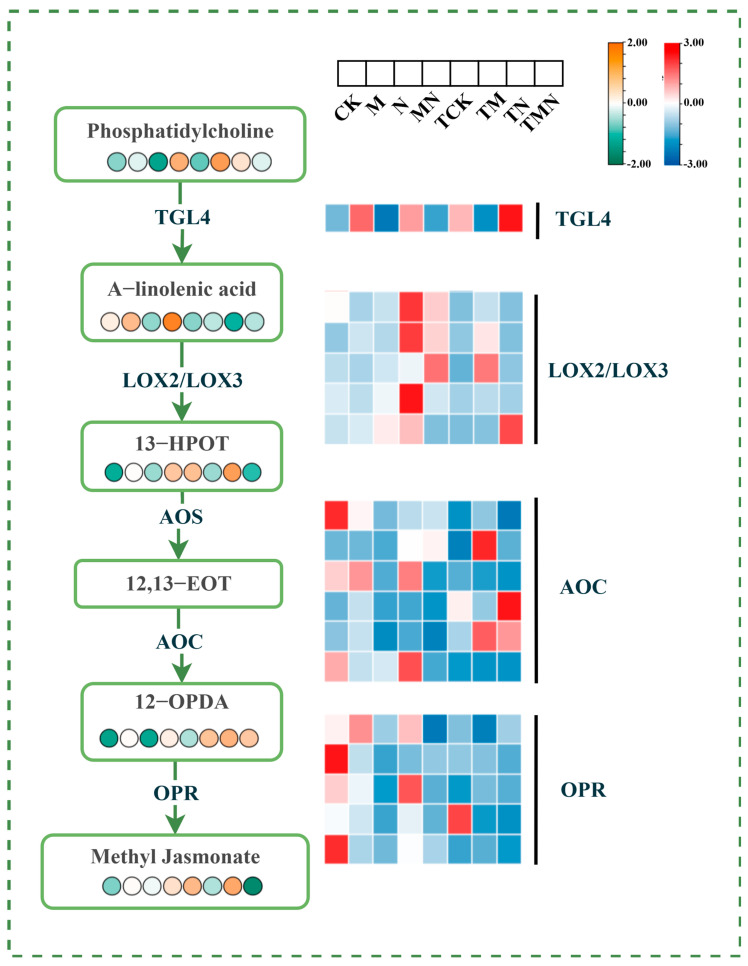
Integrative omics reveals MeJA-triggered potentiation of α-linolenic acid metabolism under salt stress.

α-Linolenic acid (ALA), a critical polyunsaturated fatty acid, serves as the primary precursor for jasmonic acid (JA) biosynthesis. Under salt stress conditions, exogenous methyl jasmonate (MeJA) application significantly modulated the α-linolenic acid metabolic pathway. Compared to the N treatment, the MN group exhibited marked upregulation of genes encoding key JA synthesis enzymes: TGL4, LOX2/LOX3, AOC, and OPR at 1 day post-treatment. No significant changes were observed at 7 days post-treatment. Additionally, metabolites within the JA synthesis pathway, including phosphatidylcholine (a lipid signaling molecule), α-linolenic acid, 13-(S)-hydroxyoctadecatrienoic acid (13-HOT), and jasmonic acid, were significantly elevated at 1 day post-treatment (Figure 9).

## 4. Discussion

### 4.1. MeJA Mitigates Salt Stress and Enhances Plant Growth

Salt stress significantly inhibits walnut seedling growth by inducing osmotic imbalance, ion toxicity, and reactive oxygen species (ROS) bursts, leading to reduced biomass and leaf water content (Figure 1). This pattern aligns with the typical damage caused by plant salt stress [19]. This study found that exogenous methyl jasmonate (MeJA), the active form of jasmonic acid signaling molecules [20], effectively mitigated this damage [21]. Its core mechanism involves synergistically activating a multi-level antioxidant defense network: physiologically, MeJA significantly increased plant biomass and relative leaf water content under salt stress. Molecularly, MeJA treatment for 1 day rapidly induced the upregulation of antioxidant enzyme genes (SOD, POD, APX, GPX, and DHAR). This directly drove enhanced SOD, POD, and APX enzyme activity while simultaneously reducing malondialdehyde (MDA) content, a product of membrane lipid peroxidation. Furthermore, MeJA enhanced antioxidant signal transduction by activating key transcription factor families such as NAC, MYB, ERF, and HSF. By day 7, expression of these enzyme genes and transcription factors decreased, accompanied by reduced soluble sugar (SS) content, suggesting a gradual stabilization of the stress response.

Photosynthetic damage is a key consequence of salt stress-induced reactive oxygen species (ROS) accumulation [22]. Photosynthetic pigments, essential for light energy capture and conversion, directly determine photosynthetic capacity [23]. Salt stress reduces photosynthetic pigment content by inhibiting chlorophyll biosynthesis and impairs water transport efficiency and redox activity. These combined effects cause a decline in photosynthetic efficiency [24]. Consistent with findings in walnuts by Li et al. [25], this study confirmed that salt stress significantly reduced chlorophyll content and photosynthetic performance in walnut seedlings. Exogenous MeJA application increased chlorophyll content, key photosynthetic parameters (net photosynthetic rate, *P*_n_; stomatal conductance, *G*_s_; transpiration rate, *T*_r_), and PSII maximum photochemical efficiency (*F*_v_/*F*_m_) under salt stress. Increased expression of key chlorophyll biosynthesis genes (HEMB, UROS, UROD, CHLI) was observed (Supplementary Figure S4A), aligning with reports of MeJA pretreatment enhancing these genes in ryegrass [26]. Furthermore, transcriptomic analysis revealed that short-term MeJA treatment upregulated genes encoding PSII components, such as *PsbB* (Figure S4B), mirroring findings where MeJA enhanced photosynthesis under cold stress via increased Psb27 and OEE3 expression [26,27]. Over longer durations, the expression of photosynthesis-related genes stabilized.

### 4.2. Phenylalanine Metabolism Mediates MeJA-Induced Antioxidant Defense

The phenylalanine metabolic pathway is crucial for the methyl jasmonate (MeJA)-mediated plant antioxidant defense system. As a key secondary metabolism precursor, phenylalanine serves as the foundation for lignin synthesis [28] and initiates flavonoid biosynthesis via catalysis by key enzymes like phenylalanine ammonia-lyase (PAL). Combined transcriptomic and metabolomic analyses revealed that exogenous MeJA (MN treatment) significantly enhanced phenylalanine metabolism in salt-stressed walnut plants. This enhancement occurred through MeJA-induced upregulation of key phenylalanine pathway genes (e.g., PAL, 4CL), driving the biosynthesis of protective secondary metabolites, notably flavonoids and lignin.

In flavonoid synthesis, phenylalanine is converted by PAL, C4H, 4CL, and chalcone synthase (CHS) into naringenin chalcone, which is then transformed into various flavonoid classes by enzymes including chalcone isomerase (CHI) [29,30]. We found that MN treatment significantly upregulated key flavonoid synthesis genes (PAL, 4CL, CHS, CHI), consistent with *Cassia obtusifolia* L. studies [30]. Furthermore, downstream genes such as flavanone synthase (FLS), flavonoid 6-hydroxylase (F6H), and dihydroflavonol reductase (DFR) were also upregulated, accompanied by significant flavonoid metabolite accumulation in the MN group. Consequently, MeJA enhances the synthesis and accumulation of these antioxidant secondary metabolites by synchronously activating flavonoid and lignin pathways. This cascade resulted in more efficient reactive oxygen species (ROS) scavenging, reducing membrane lipid peroxidation and providing a key metabolic mechanism for walnut seedlings to mitigate salt stress-induced oxidative damage.

### 4.3. Tryptophan Metabolism Mediates MeJA-Induced Antioxidant Defense

The tryptophan metabolic pathway contributes distinctly to methyl jasmonate (MeJA)-mediated antioxidant defense. This study revealed that under salt stress, MeJA (MN) upregulated key genes in the tryptophan pathway, including tryptophan decarboxylase (DDC) and acetylserotonin methyltransferase (ASMT), enhancing melatonin synthesis. Melatonin operates dually in this system: (1) As a potent non-enzymatic antioxidant, it directly scavenges reactive oxygen species (ROS); (2) MeJA induces the activation of essential antioxidant enzymes, including superoxide dismutase (SOD) and peroxidase (POD), forming a protective enzymatic barrier against oxidative damage. This dual functionality significantly boosts the overall antioxidant capacity of walnut seedlings.

Furthermore, the tryptophan pathway indirectly bolsters the antioxidant network by regulating auxin (indole-3-acetic acid, IAA) homeostasis. MeJA treatment upregulated tryptophan aminotransferase (TAA1) gene expression, optimizing the IAA synthesis pathway and maintaining IAA levels essential for cell division and elongation. Maintaining IAA homeostasis provides cellular and metabolic resources for synthesizing protective metabolites, including antioxidants [31]. Thus, MeJA promotes melatonin accumulation—possessing dual antioxidant functions—through the tryptophan network, while concurrently supporting antioxidant defense via IAA stability. The synergy between melatonin and IAA not only reduces ROS accumulation and membrane lipid peroxidation but also balances antioxidant defense with growth processes, representing a key mechanism for walnut seedlings to mitigate salt stress—induced oxidative damage.

### 4.4. MeJA Alleviates Salt Stress Through the α-Linolenic Acid Metabolism Pathway

As an important unsaturated fatty acid in plants, α-linolenic acid is not only the starting substrate for jasmonic acid (JA) biosynthesis but also plays a central role in plant stress signaling. JA, as a classical signaling molecule, participates in the plant salt stress response by coordinating ionic homeostasis and the antioxidant defense system. Numerous studies have shown that exogenous JA and its derivative MeJA can enhance plant tolerance to a wide range of abiotic stresses. For example, Sánchez-Martín et al. [32] found that MeJA treatment increased levels of JA-Ile, JA, and MeJA under drought stress. In this study, based on joint transcriptome and metabolome analysis, exogenous MeJA treatment significantly elevated jasmonic acid and α-linolenic acid metabolism levels in walnut under salt stress. At the molecular level, MeJA up-regulated the expression of genes encoding key enzymes for jasmonic acid synthesis, such as TGL4, LOX2, and LOX3, and activated the activities of LOX, AOC, and OPR. These reactions promoted jasmonic acid biosynthesis and alleviated salt damage through two mechanisms: inhibiting LOX-mediated lipid peroxidation and stabilizing the photosynthetic membrane structure. This led to efficient ROS scavenging, maintenance of membrane stability, and enhanced salt tolerance in the plant.

While this study provides comprehensive mechanistic insights, key limitations warrant acknowledgment: First, findings derive exclusively from seedling-stage walnuts under controlled conditions. Physiological and molecular responses in mature orchard trees may diverge due to field-environment interactions (e.g., rhizosphere microbiome, diurnal climate fluctuations). Second, the transcriptomic and metabolomic analyses captured only two timepoints (1d/7d). Higher-resolution temporal profiling could reveal critical transient regulatory events during early stress adaptation and compensatory mechanisms during sustained stress.

## 5. Conclusions

This study integrated physiological, biochemical, and multi-omics analyses to demonstrate that exogenous methyl jasmonate (MeJA) effectively mitigates salt stress-induced growth impairment in walnut seedlings. The core mechanism involves MeJA-mediated synergistic activation and the coordination of antioxidant defense and photosynthetic systems, significantly maintaining cellular homeostasis to enhance salt tolerance. Transcriptomic and metabolomic analyses revealed pathway-specific regulatory roles: In phenylalanine metabolism, MeJA upregulated key genes (PAL, 4CL), promoting lignin and flavonoid accumulation to strengthen cell walls and enhance ROS scavenging efficiency; in tryptophan metabolism, MeJA elevated the expression of DDC and ASMT genes, stimulating auxin biosynthesis, direct ROS scavenging, and activation of enzymatic antioxidant systems (e.g., SOD/POD); in α-linolenic acid metabolism, MeJA primarily enhanced JA-Ile biosynthesis and signaling, thereby inhibiting lipid peroxidation and stabilizing membrane structures. Consequently, this work elucidates the physiological and molecular networks through which MeJA coordinates key metabolic pathways to enhance salt stress resilience. It provides a theoretical foundation for improving stress tolerance in woody economic species and supports developing MeJA-based agronomic strategies for saline soils. Future research should prioritize the following: (1) Field validation trials of foliar-applied MeJA (50–100 μM) in saline-affected walnut orchards across major production regions (e.g., Xinjiang, California) to optimize application protocols and salinity range adaptation; (2) Elucidation of metabolic crosstalk between JA signaling and key phytohormones (ABA, ETH) under combined stress scenarios; (3) Extension of the MeJA-mediated salt tolerance framework to other economically significant perennial woody through comparative transcriptomics and physiological screening.

## Figures and Tables

**Table 1:** Different concentrations of walnut seedlings.

Treatment (1d)	Treatment (7d)	NaCl Concentration (mM)	MeJA Concentration (μM)
CK	TCK	0	0
M	TM	0	50
N	TN	50	0
MN	TMN	50	50

Abbreviations: CK: Control (no stress, Day 1); TCK: Time-point control (no stress, Day 7); M: 50 μM MeJA treatment (no NaCl, Day 1); TM: 50 μM MeJA treatment (no NaCl, Day 7); N: 50 mM NaCl stress (no MeJA, Day 1); TN: 50 mM NaCl stress (no MeJA, Day 7); MN: 50 mM NaCl + 50 μM MeJA (Day 1); TMN: 50 mM NaCl + 50 μM MeJA (Day 7). Concentrations: All numerical values in NaCl and MeJA columns represent actual treatment concentrations.

## Data Availability

Data are contained within the article.

## Supplementary Materials

The following supporting information can be downloaded at: https://www.mdpi.com/article/10.3390/antiox14080974/s1, Figure S1: Enrichment analysis of the KEGG pathway; Figure S2: (A) DEGs involved in chlorophyll biosynthesis. (B) DEGs involved in photosynthesis; Figure S3: RT-qPCR was performed to verify the expression pattern of key genes in walnut under different treatments. Values presented are mean ± SD (n ≥ 3), are the relative expression levels of 6 genes, and their abscissas are the corresponding genes. Bars represent the results of RT-qPCR and lines represent the results of RNA-Seq. The scale on the left axis represents relative expression, and the right axis represents FPKM values; Table S1: Primer sequences for qRT-PCR; Table S2: The effects of different MeJA concentrations on the growth of walnut.
