# Supplementary material for: Methyl Jasmonate Orchestrates Multi-Pathway Antioxidant Defense to Enhance Salt Stress Tolerance in Walnut (Juglans regia L.)
Source: Antioxidants (Basel). 2025 Aug 8;14(8):974. doi: 10.3390/antiox14080974 (PMC12382770; doi:10.3390/antiox14080974)
Supplement: Supplementary file 1 [file antioxidants-14-00974-s001.zip › antioxidants-3777141-supplementary.pdf]

**Table S1.** Primer sequences for qRT-PCR.

| Gene name | Forward primer sequence (5’-3’) | Forward primer sequence (5’-3’) |
|---|---|---|
| JrMYB1 | TTCCCAAAAGAGCAGGGCTTA | CATTGTCTGTTCGGCCTGGA |
| JrMYB7 | CCCAAAAAGGCTGGGCTCC | CATTGTCAGTACGGCCAGGA |
| JrC3 | GAGATTCAACAAGGCGTGGC | CGCACAGCCATACTTCATGC |
| JrIAA14 | CCACCTGCCAAGGCACACA | AGGCATCGGATAGCTCTTGG |
| JrPER4 | GCCGATAGCAAGCTTTCCC | CCGATGCGTCACAGCCATTTA |
| JrPERP7 | TCGCTTGTTCTTCCACGACT | TGGGGATCCTTTCAGCAAGAC |

**Table S2.** The effects of different MeJA concentrations on the growth of walnut.

| Treatment | SPAD | Tchl | MDA | SS |
|---|---|---|---|---|
| Control | 32.57±2.25a | 3.54±0.14a | 58.47±10.12c | 61.71±1.75ab |
| NaCl | 26.67±2.06abc | 3.31±0.01b | 73.64±1.74a | 63.78±2.11a |
| M50 | 30.87±0.81ab | 3.52±0.07a | 60.61±1.64bc | 66.01±5.80a |
| M100 | 28.17±4.22abc | 3.28±0.15b | 64.72±3.00abc | 64.01±1.51a |
| M200 | 25.47±2.51bc | 2.66±0.04c | 64.29±0.92abc | 67.10±2.77a |
| M400 | 24.33±5.01c | 2.53±0.01c | 70.32±6.12ab | 55.74±4.78b |

The values are represented as average ± standard deviation. Letters indicate significant differences ($p<0.05$) via Tukey’s test. Treatments: Control: 1/2 Hoagland’s solution; NaCl: Control + 50 mM NaCl; M50: Control + 50 μM MeJA + 50 mM NaCl; M100: Control + 100 μM MeJA + 50 mM NaCl; NM200: Control + 200 μM MeJA + 50 mM NaCl; NM400: Control + 400 μM MeJA + 50 mM NaCl

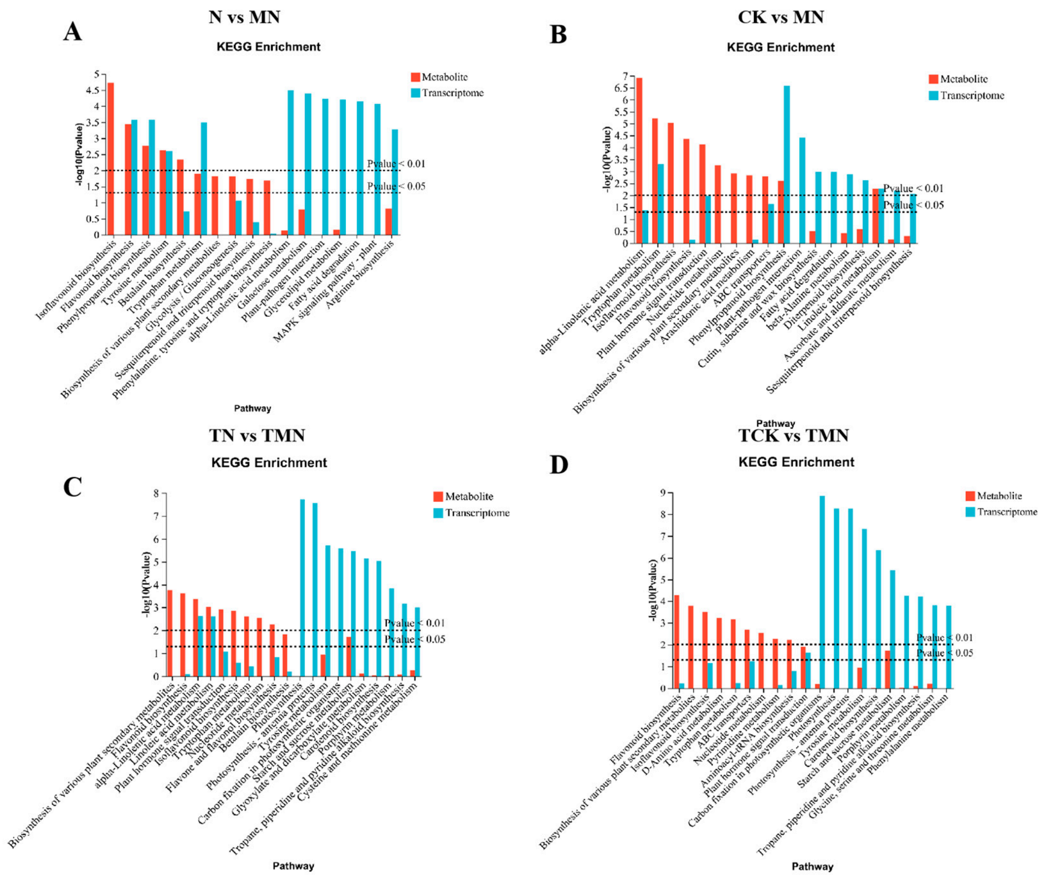


**Figure S1.** Enrichment analysis of the KEGG pathway.

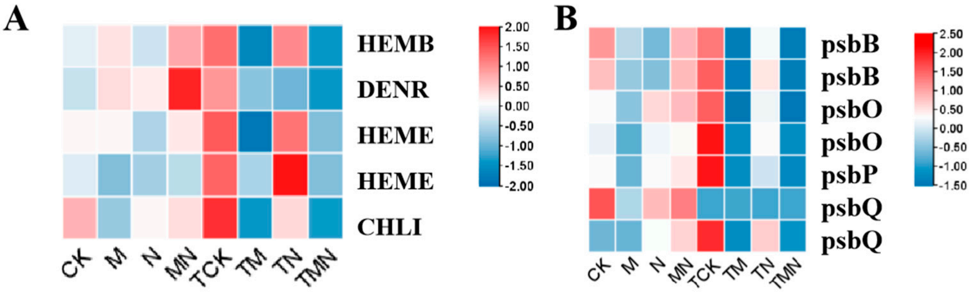


**Figure S2.** (A) DEGs involved in chlorophyll biosynthesis. (B) DEGs involved in photosynthesis.

*qRT-PCR validation of the transcriptome data*

The expression patterns of 6 selected DEGs were evaluated in CK, M, N, MN, TCK, TM, TN, and TMN by RT-qPCR using gene-specific primers to validate the accuracy and reliability of RNA-Seq data. The comparative results demonstrated that the expression patterns of the 6 genes identified by RT-qPCR were consistent with those from RNA-Seq sequencing, further confirming the reliability of transcriptome data (Fig. S3).

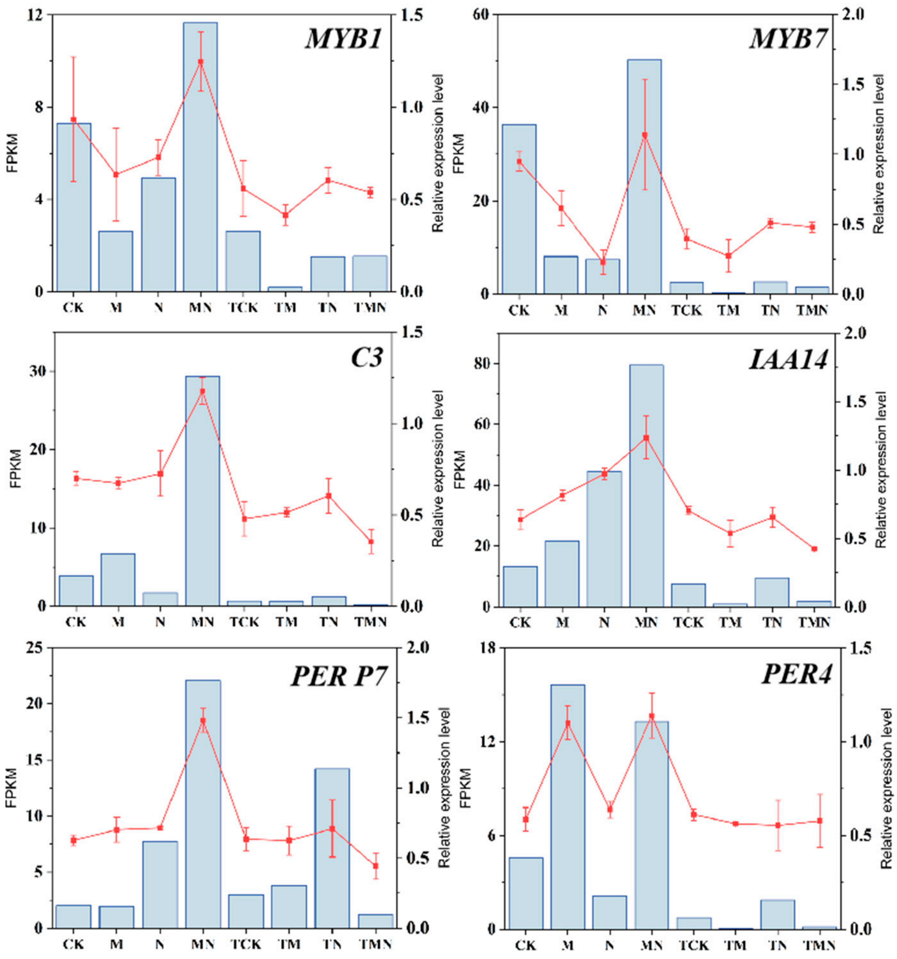


**Figure S3.** RT-qPCR was performed to verify the expression pattern of key genes in walnut under different treatments. Values presented are mean ± SD (*n*≥3), are the relative expression levels of 6 genes, and their abscissas are the corresponding genes. Bars represent the results of RT-qPCR and lines represent the results of RNA-Seq. The scale on the left axis represents relative expression, and the right axis represents FPKM values.
